# *Vitis vinifera* L. cv. Falanghina Seed Extracts: Antioxidant Effect of Bioactive Compounds on HepG2 Cells

**DOI:** 10.3390/antiox13070834

**Published:** 2024-07-12

**Authors:** Stefania Iervolino, Pierpaolo Scarano, Jessica Raffaella Madera, Cristina Franco, Maria Tartaglia, Romania Stilo, Rosaria Sciarrillo, Lorella Maria Teresa Canzoniero, Maria Moreno, Carmine Guarino

**Affiliations:** 1 Department of Neurosciences, Reproductive Sciences and Dentistry, University of Naples ‘Federico II’, Via Pansini, 5, 80131 Naples, Italy; stefania.iervolino@unina.it; 2 Department of Science and Technology, University of Sannio, Via F. de Sanctis, snc, 82100 Benevento, Italy; jrmadera@unisannio.it (J.R.M.); cfranco@unisannio.it (C.F.); mtartaglia@unisannio.it (M.T.); romstilo@unisannio.it (R.S.); sciarril@unisannio.it (R.S.); canzoniero@unisannio.it (L.M.T.C.); moreno@unisannio.it (M.M.); guarino@unisannio.it (C.G.)

**Keywords:** Soxhlet extraction, UHPLC, HRMS, GC-FID, total phenolic compounds, radical scavenging activities, valorization, cytocompatibility, mitochondria

## Abstract

*Vitis vinifera* L. is a natural source of bioactive compounds that is already used for cosmeceutical and nutraceutical approaches. However, their phytochemical and antioxidant properties, although studied, have not been fully explored. We aimed to characterize *V. vinifera* L. cv. Falanghina seed extracts in different polarity solvents (hexane, ethyl acetate, ethanol, and a mixture of acetone–water) for their phytochemical contents, including the total phenolic compound content (TPC), free radical scavenging capacities, and antioxidant ability on HepG2 cells. We directly profiled the functional quality of *V. vinifera* seed extracts against H_2_O_2_-induced oxidative stress in HepG2 cells, focusing on mitochondrial functions. The content of bioactive compounds was characterized by LC-MS. To assess the cytocompatibility of the extracts, a 3-(4,5-dimethylthiazol-2-yl)-2,5-diphenyltetrazolium bromide (MTT) assay was conducted. Results showed that extraction with ethyl acetate (18.12 mg GAE·g^−1^) and ethanol solvents (18.07 mg GAE·g^−1^), through Soxhlet, and with an acetone–water mixture (14.17 mg GAE·g^−1^), through maceration, yielded extracts rich in (poly)phenols, with good scavenging and antioxidant activity (98.32 I% for ethanol solvents and 96.31 I% for acetone–water mixture). The antioxidant effect of polyphenols is at least partially due to their capacity to maintain mitochondrial biogenesis and mitophagy, which elevates mitochondrial efficiency, resulting in diminished ROS production, hence re-establishing the mitochondrial quality control. These findings highlight the valorization of *Vitis* by-products to improve food functional characteristics.

## 1. Introduction

Many bioactive compounds in nature can positively and negatively affect cellular functions [1,2]. Phenolic compounds are recognized for having high antioxidant activity due to their redox properties [3,4], and play important roles in absorbing and neutralizing free radicals [5].

Plants such as the grapevine (*Vitis vinifera* L.), which is rich in bioactive compounds, are widely used for developing products used in various sectors, such as pharmaceuticals, nutraceuticals, and cosmeceuticals [6]. These compounds are present in all parts of the plant, that is, in the berries, consisting of skin, pulp, and seeds; in the stalks (the woody part attached to the berries that, when wine is made, is removed as waste); in the leaves; and in the roots [7,8,9,10,11,12]. According to what is happening at the EU and at a world regulatory level in the reduction of waste, its management, and, above all, its valorization, every secondary part derived from primary production (the waste) should be ennobled. In the case of the grapevine, this is already happening in part, as waste from wine production is diverted to the production of grappa, according to an elective production process [13,14]. Nonetheless, it would be interesting to improve the use of such a waste since it contains large quantities of bioactive compounds of interest [13]. In parallel, the development of new technologies that allow the recovery of waste and, in particular, the bioactive compounds contained within, would support the production of products of interest to the cosmeceutical and even the nutraceutical and pharmaceutical industries [15].

The grape is the berry produced by the plants of the genus *Vitis*, which includes approximately 60 different species of vines: *Vitis vinifera* L. is the most cultivated species (90% of the berries on the market). Grapes are one of the most popular fruit crops in the world, with approximately 77.44 million tons of production in 2016 [16]. The reason for the prevalence of such a wide range of processed products is due to the extreme perishability of the fruit [17]. The waste from processed grapes (grape seeds and grape skins) can be recovered for further food applications. They are rich in cellulose (20.8%), hemicelluloses (12.5%), proteins (18.8%), tannins (13.8%), extracts soluble in dichloromethane (5.0%), and ash (7.8%). The water-soluble compounds (26.4%) are mainly composed of monomeric sugars (glucose and fructose) and a complex mixture of hemicelluloses, with pectin and acetylated glucomannan being the most abundant [13]. The predominant bioactive compotes are represented by the content of polyphenols (anthocyanins and tannins), which generally influences both the sensorial characteristics and the healthy properties of the wine as a product [18,19]. Among all the compounds, the presence of resveratrol, a non-flavonoid compound with important medical characteristics, stands out [20,21,22]. The anthocyanins normally present are the 3-O-monoglucosides of malvidine, delphinidine, peonidine, petunidine, and cyanidine, together with the corresponding derivatives of acetyl, p-coumaryl, and caffeoile [7,9]. From grape waste, therefore, it is possible to recover phenolic compounds such as flavonoids, especially present in grape seeds, which have a high antioxidant activity important for their pharmacological properties, while resveratrol has proven to be an antifungal, anti-inflammatory, and anticarcinogen [23,24]. It has been shown that there is an important link between the bioactivity of certain compounds listed above and several non-polar, such as lipids, and semipolar compounds, such as (poly)phenolic molecules, which are highly accumulated in grape seeds [25].

Several studies have described the antioxidant effects of molecules extracted from grape tissues [5,26,27,28]. Since oxidative stress is a byproduct of mitochondrial activity, the antioxidant effects could be due to the involvement of the mitochondrial compartment. However, no studies have so far looked at mitochondria directly. Moreover, mitochondrial functional analysis has recently been reported as a new tool to determine the quality of plant-extracted products [29]. Indeed, due to their role in cellular metabolism, as well as in several cellular activities, assessing mitochondrial function is more closely linked to the biological activity of products which can be characterized for their potential clinical effectiveness. The aim of the present study was to directly profile the functional quality of *V. vinifera* seed extracts against H_2_O_2_-induced oxidative stress in HepG2 cells, focusing on mitochondrial functions. Although, not used to represent hepatocytes, HepG2 cells were employed as an in vitro model to investigate cytotoxicity in general [30]. Furthermore, due to their high contents of organelles, HepG2 cells represent an established model for investigating mitochondrial parameters [31].

## 2. Materials and Methods

### 2.1. Chemicals and Reagents

Microfiltered and ultrapure water was used for the preparation of the solutions via the Merck Millipore ZRQS0P3FR Direct system (MilliporeSigma, Burlington, MA, USA). All solvents and reagents used in the experiments were of a high degree of purity and are reported as follows: ethanol ≥ 99.9% ACS for analysis, methanol for HPLC, anhydrous 95% n-hexane, chloroform for chromatography, 1-butanol ACS reagent ≥ 99.5%, hydrochloric acid 37% RPE for analysis, potassium bicarbonate, anhydrous sodium carbonate for analysis, gallic acid ACS for analysis, and Sudan IV reagent were purchased from Sigma-Aldrich Chemical Company (Milan, Italy); Folin–Ciocȃlteu reagent and hypergrade acetonitrile for LC-MS were purchased from Merck Millipore GmbH (Milan, Italy); polyphenol standards (malvidin-3-O-glucoside, naringin, catechin, quercetin, gallic acid, vanillic acid, caffeic acid, and ferulic acid), methanol, and acetic acid were purchased from Merck (Darmstadt, Germany); 2-propanol and acetonitrile (ACN) were purchased from Honeywell (Charlotte, VA, USA); formic acid was obtained from J.T. Baker (Rodano, Italy); and 2,2-diphenyl-1-picrylhydrazyl (DPPH) was obtained from Alfa Aesar (from Thermo Fisher Scientific companies in Rodano, Milan, Italy). Unless otherwise indicated, all culture materials were purchased from Gibco-Invitrogen (Carlsbad, CA, USA). Extracts obtained from *V. vinifera* L. cv. Falanghina (seed from fruits) to be analyzed were prepared by diluting the stock solutions of the extracts in water or a hydroalcoholic solution.

### 2.2. Plant Materials

Vegetal materials of *V. vinifera* L. cv. Falanghina (seed from fruits), sampled during the fruiting period of the plant, were collected between September and early October 2022. The sampling points were in Southern Italy (41°14′07.78″ N, 14°33′32.39″ E, known as Telesina Valley, in the Campania region).

The sampling sites were in vineyards found in different areas of the municipality of Guardia Sanframondi in the province of Benevento, 100–500 m a.s.l. (above sea level).

Sampling was carried out by collecting a quantity of matrices (fruits, from which seeds were then obtained) equal to 500 g on different plants of the species, present in different vineyards, to minimize intraspecific variability and obtain a sufficient sample to perform each type of analysis in triplicate. *V. vinifera* samples were vacuum sealed directly into clean polyethylene bags and stored in refrigerated boxes at 5 °C; they were brought to the laboratory a maximum of 2 h after collection. Subsequently, all samples were left clean and frozen at −80 °C until extraction and subsequent analysis.

### 2.3. Preparation of V. vinifera L. cv. Falanghina Extracts

Before being frozen at −80 °C, the samples were thoroughly washed with distilled water to remove parts that were not part of the sample, such as pulp residue. Each sample was then ground through a blender to obtain an extractable sample. All samples were subjected to grinding in the presence of liquid nitrogen through a Waring blender.

### 2.4. Extraction

Plant samples were extracted using the Soxhlet extraction (SE) technique. The experiments were carried out in triplicate, by preparing a quarter of the plant sample that was divided into three aliquots, which were subsequently used for the extraction process; different solvents were used, in sequence, to obtain samples with different bioactive composition: hexane (henceforth HE), ethyl acetate (henceforth EA), and ethanol (henceforth ET). After the SE extraction, a maceration extraction (ME) was conducted on the same sample by using a solution of water and acetone in a 6:4 (*v/v*) ratio (henceforth WA). Another ME with a solution of ethanol and ethyl acetate in an 8:2 (*v/v*) ratio (henceforth AE) was conducted. This extraction was conducted using a methodological approach already found in the literature [32], which was suitably modified to improve it and to compare it with our methodology. In particular, a solvent such as ethyl acetate was used instead of chloroform and the amount of initial plant matrix was considered to enhance the quantitative results.

The SEs were carried out at reflux, with boiling solvent, at relative times due to depletion of the matrix in the extractable components compared to the solvent used, but not exceeding 24 h. Extractions were conducted by keeping the ratio of matrix to extracting solvent constant (1:10), generally by feeding 10 g of matrix with 100 mL of solvent into the Soxhlet apparatus. The ME was performed with the same matrix–solvent ratio at room temperature. The extracted samples were subjected to desolvation by using a rotary evaporator (HEIDOLPH Heizbad Hei-Vap from Heidolph Instruments GmbH & Co. KG, based in Schwabach, Germany): the evaporation temperature of the bath was set between 35 and 40 °C. Subsequently, extracted samples were redissolved for analysis. In Table 1, a list of extract samples obtained and subjected to characterization is reported. Each sample was associated with a unique code.

Several analytical parameters were quantified for all crude extracts: soluble solid residue, dry residue, and the amount of extracted sample per amount of extracted matrix. Parameters such as dry residue and total extraction yield (Residue and Extract, respectively, in Table 2) were reported for each extract obtained with the different techniques and solvents. The dry residue allows one to extrapolate the amount of sample extracted from the matrix per amount of solvent used (expressed in g·mL^−1^), thus allowing one to calculate the extraction yield for the type of solvent used, depending on the technique. The total extraction yield calculates the maximum amount of extract obtained per amount of matrix used, revealing the maximum amount of substance that can be extracted with that specific method under the listed conditions (expressed in $mg\cdot g_{seed}^{-1}$ dried weight). In addition, in Table 2, the °Bx parameter (tabulated by NIST) [33] is explained to evaluate the specific gravities that represents the dissolved sugar content, which was calculated from the percentage sugar content (dissolved solids content) with the Brix and Gravity Refractometer (HI96801 portable refractometer from Hanna Instruments Italia Srl, based in Padova, Italy), with automatic temperature compensation (ATC) that features a 0.0–85.0%.

All extract samples were solubilized in double-distilled H_2_O with 5% absolute EtOH. The remaining non-solubilized part was removed from the sample by filtration and was not used in subsequent analyses and in vitro studies.

### 2.5. Total Phenolic Compound Content (TPC)

The content of total phenolic compounds was measured according to the Folin–Ciocȃlteu reagent method [34], with some minor modifications following Scarano et al., 2022 [35]. The total content of phenolic compounds was expressed in gallic acid equivalents (GAE): the data were expressed as the mg of GAE per g of extract expressed in mg GAE∙g^−1^ using the calibration curve of gallic acid standard solutions (50–250 mg∙L^−1^). All measurements were taken in triplicate and calculated as mean value ± SD (n = 3).

The absorbance measurements for the TPC and for DPPH assay analysis were performed by a MERCK (Milano, Italy) Spectroquant^®^ Pharo 300 UV/Vis spectrophotometer, by using a 1.0 cm-long optical path glass cell.

### 2.6. 2,2-Diphenyl-1-picryl-hydrazyl-hydrate (DPPH) Antioxidant Assay (DPPH) Radical Antioxidant Assay

The antioxidant activity of the extracts was estimated by using the DPPH assay according to the modification of the method made in Scarano et al., 2022 [35], reported in Mosquera et al., 2007 [36].

Absorption of the samples at 517 nm was determined spectrophotometrically and the radical scavenging activity was then expressed as the percentage of free radical inhibition by the sample and was calculated and expressed as % free radical inhibition (I%) (1). The I% is directly related to the antioxidant power of the sample. The I% was calculated according to the following equation:

(1) $$I\%=\left[\frac{\left(A_{control}-A_{sample}\right)}{A_{DPPH}}\right]\times 100,$$

where *A_sample_*, *A_control_*, and *A_DPPH_* are the absorbance of the sample, absorbance of the control, and absorbance of the DPPH solution, respectively. Trolox was used as the positive control. All samples were prepared at a concentration of 100 μg·mL^−1^.

### 2.7. UHPLC/UV-ESI-HRMS Analysis

UHPLC/UV-ESI-HRMS analyses were conducted by using Dionex Ultimate 3000 RS, Thermo Scientific (Rodano, MI, Italy), equipped with a Hypersil Gold C18 column (100 × 2.1 mm, 1.9 μm particle size, Thermo Scientific), coupled to a Q-ExactiveTM high-resolution mass spectrometer (Thermo Scientific, Rodano, MI, Italy).

The chromatographic column was equilibrated in 98% solvent A (0.1% aqueous solution of formic acid) and 2% solvent B (methanol) for 0.5 min. The flow rate in the column was maintained at 300 μL∙min^−1^ and the concentration of solvent B was increased linearly from 2% to 23% in 5.5 min, remaining in isocratic for 5 min, then increased linearly from 23% to 50% in 7 min, and from 50% to 98% in 5 min, remaining in isocratic for 6 min, and finally returned to 2% in 6 min, remaining in isocratic for 3 min. The UV/VIS detector was set at 235, 330, 480, and 535 nm. The volume of sample injected was 5 μL.

Electrospray (ESI) in negative polarity was selected for mass analysis, with the following operating conditions: resolution: 70,000 (FWHM at *m*/*z* 200); IT 100 ms; ACG target = 1 × 10^6^; scan range (100–1000 *m*/*z*). In each scan, the most intense precursors were automatically selected by the instrument and MS/MS analysis was performed on them with the following operating conditions: resolution: 35,000; AGC target = 1 × 10^5^; maximum IT 200 ms; collision energy (stepped NCE): 25, 30, 40. The quadrupole isolation window was set to 1.6 *m*/*z*. The instrument was calibrated before each analysis using the calibration solution supplied by Thermo Fisher Scientific.

Prior to analysis, 1 mg of samples were diluted in 5% EtOH to a concentration of 1 mg·mL^−1^. The samples analyzed are listed in Table 1. The analytes’ composition was reported as the relative area percentage (%) of total peaks.

### 2.8. GC/FID Analysis

Fatty acid methylation [37] was performed via a base-catalyzed transesterification procedure to avoid isomerization, as reported by Sassano et al., 2009 [37]. Briefly, 20 mg of each sample was added to a vial along with 1 mL of 0.5 M sodium methoxide solution. The vial was placed in a thermostatted water bath (70 °C for 10 min) to allow for complete methylation. At the end of the reaction, the vial was cooled and 4 mL of isooctane was added under stirring to extract the fatty acid methyl esters (FAMEs). Then, 6 mL of deionized water was added and the isooctane layer was collected and dried with Na_2_SO_4_. After centrifugation, the organic phase was collected and analyzed. Appropriately, FAMEs were analyzed by GC-FID using Trace GC Ultra, Thermo Scientific (Rodano, MI, Italy), equipped with an RT-2650 column (100 m × 0.25 mmID, 0.20 μm, Restek, Centre County, PA, USA). The injector and detector temperatures were 240 °C and 250 °C, respectively. The split ratio was 50:1. The temperature ramp used was as follows: initial temperature 100 °C held for 3 min; increase of 3 °C/min to 200 °C for 3 min; increase of 3 °C/min to 240 °C for 12 min. The total run time was 65 min. Fatty acids were recognized using the Supelco 37 component FAME Mix (Merck KGaA, Darmstadt, Germany) standard. The volume of sample injected was 1 μL. Fatty acid composition was reported as relative area percentage (%) of total peaks.

### 2.9. Cell Culture

Human hepatoma cells (HepG2) were provided by Prof. Dr. Pieter De Lange from the University of Campania Luigi Vanvitelli. HepG2 cells were cultured in Dulbecco’s Modified Eagle’s Medium (DMEM) supplemented with 10% *v/v* fetal bovine serum (FBS) and 1% *v*/*v* penicillin, 1% *v*/*v* streptomycin in 100 mm tissue culture Petri dishes at 37 °C in a humidified 5% CO_2_ incubator. Cells were fed every 2–3 days and were subcultured once they reached 70–80% confluence.

Cells were seeded in 96-well plates at a density of 2 × 10^5^ cells·mL^−1^ per well or on 11 mm glass coverslips coated with 10 µg·mL^−1^ poly(L)-lysine (Sigma-Aldrich). After 24 h, the culture medium was removed and equal volumes (100 μL) of medium with increasing concentrations (25 g·mL^−1^, 50 g·mL^−1^, 75 g·mL^−1^, and 100 g·mL^−1^) of *V. vinifera* L. seed extracts dissolved in 5% H_2_O/ethanol were administered for 24 h, 48 h, and 72 h. Control cells received the same medium without extract and with the same volume of 5% H_2_O/ethanol. Under the same conditions, cells were treated with increasing concentrations of H_2_O_2_ (100 µM, 300 µm, 500 µM, and 1000 µM) for 3, 6, and 24 h, respectively. In the post-treatment experiments, cells were treated with seed extracts for an additional 3 h after the 3 h H_2_O_2_ treatment. At the end of the treatment, the cells were used to detect intracellular ROS production or to evaluate protein expression.

### 2.10. MTT Assay

To evaluate the effects of *V. vinifera* L. seed extracts on HepG2 mitochondrial function, the 3-[4,5-dimethyltiazol2yl]-2,5-diphenyltetrazolium bromide (MTT) assay was used. At the end of each assay period, cells were incubated in the dark with 10 μL of 5 mg·mL^−1^ MTT for 2 h at 37 °C in 5% CO_2_, and formazan crystals were carefully dissolved with 100 μL isopropanol for 15 min. The absorbance was measured at 570 nm on a microplate spectrophotometer (Infinite 200 Pro 200, Tecan, Männedorf, Switzerland). Data were expressed as a percentage of the absorbance of the untreated cells. Plots are representative of six replicates and error bars are calculated as the standard deviation.

### 2.11. Measurements of Intracellular ROS (DCFH-DA)

The formation of intracellular ROS was detected using the cell membrane permeable fluorescein analogue 2′,7′-dichlorofluorescin diacetate (DCFH-DA). The cells were grown on 11 mm glass coverslips into a 24-well plate at 6 × 10^4^ cells·mL^−1^. At the end of each treatment, cells were washed twice with PBS and then incubated in PBS containing 10 μM of DCFH-DA for 30 min at 37 °C in darkness. Coverslips were washed twice with PBS and mounted on an Olympus BX51 Fluorescence (EVIDENT CORPORATION, Tokyo, Japan) upright microscope equipped with a 20× objective, exciting samples at 485 nm for 12 s and filtering emitted light through a 530 nm barrier filter. Images were digitalized using an ORCA-spark Digital CMOS camera (Hamamatsu Photonics K.K., Tokyo, Japan). At least three images of randomly selected fields of view were used for data analysis using ImageJ software (ImageJ software avaiable at https://imagej.net/software/imagej2/ (software version 2.15.0, full access 11 July 2024, NIH, Bethesda, MD, USA)).

### 2.12. Western Blot Analysis

Total protein extracts were prepared in RIPA buffer lysis buffer containing 50 mM Tris HCl pH 8, 150 mM NaCl, 1% NP-40, 0.5% sodium deoxycholate, 1 mM EDTA, 1 mM NaF, 100 mM Na_3_VO_4_, and 0.1% SDS. Protease and phosphatase inhibitors were also added before use. Cell lysates were kept on ice for 1 h and then centrifuged at 10,000 rpm for 15 min at 4 °C. Total protein levels were determined by the Bradford assay, and 30 μg of total proteins were loaded onto a 12% sodium dodecyl sulfate–polyacrylamide gel (SDS-PAGE) and electrophorized at 200 V. After electrophoresis, proteins were transferred to nitrocellulose and immunoblotted. Membranes were incubated overnight at 4 °C with primary antibodies in 5% milk or BSA, washed with PBS-T, and exposed to alkaline HRP-conjugated secondary antibodies (1 h at RT). GAPDH was used as a loading control. Immunoreactive bands were visualized by chemiluminescence using the ChemiDoc MP system (Biorad, Hercules, CA, USA) and the signal was quantified using Image Lab software (Image Lab software avaiable at https://www.bio-rad.com/it-it/product/image-lab-software?ID=KRE6P5E8Z (software version 6.1, full access 11 July 2024, Biorad, Hercules, CA, USA)).

### 2.13. Data Analysis

Data were shown as the mean and standard deviation, SD (triplicate measures). For normally distributed data, differences of parameters (Brix, Residue, Extract) for each solvent (HE, EA, ET, WA, and AE) between the four extraction methods (ME, SE) were detected using one-way ANOVA with Tukey’s post hoc test (*p* < 0.05).

## 3. Results

### 3.1. Chemical Analysis of V. vinifera Extracts

To evaluate the extractive performance of the obtained extracts, several parameters were evaluated. Considering the weight of the extract (i.e., quantifying the dry residue), the extractive capacity of the solvent is reported in Table 2.

The values obtained for °Bx, regarding the carbohydrate component, were higher for the extracts in SET and MWA than in SHE and SEA. The °Bx value represents a percentage value (one degree Brix corresponds to 1 part of dissolved solid substance resulting from reading the refractometer in 99 parts of solvent to obtain 100 total parts of solution), which is very low related to the remaining solid part which is contained in the sample solution. Indeed, all the solid part in solution is identified by the dry residue (Residue), which is the sum of the dissolved solid substance and possible other bioactive components.

Next, the TPC was analyzed in all samples, preliminarily assuming a higher content in the SET and MWA samples than in the SHE and SEA ones. As reported in Table 3, SEA, contrary to what was assumed, shows a TPC value comparable with samples SET and MWA.

Quantification of the TPCs showed that in the different extracts obtained with SE, there was a good content of total phenols (expressed in gallic acid equivalents∙(mg GAE·g^−1^)). The samples showed decreasing TPC as follows: MAE >> SEA ≈ SET > MWA >> SHE.

From the TPC data, the hypothesized difference between the SET and MWA samples can be seen by comparing them with the SHE samples. It should be kept in mind that the TPC content might be overestimated because the SHE sample, and especially the SEA sample, may contain different molecules (as in the case of proteins and amino acids [38,39] not analyzed in this work) that may positively respond to the assay.

All of this leads us to identify, as expected, solvents such as EA and ET as those having a better extractive capacity towards (poly)phenolic compounds compared with solvents such as WA and HE [40].

To characterize the antiradical activity of the extracts, DPPH radical scavenging activity (I%) of different seed extracts of *V. vinifera* was determined. As reported in Table 3, there was a preference for extracting molecules with antiradical activity in SET, MWA, and MAE extracts, with I% above 95% for both (98%, 96%, and 96%, respectively) compared to the positive control, Trolox, with I% of 95%. Moderate antiradical activity was also present for SHE and SEA extracts, with percentages above 51% and 65%, respectively.

Table 4, Table 5, Table 6, Table 7 and Table 8 report the data relative to the GC-FID and UHPLC/UV-ESI-HRMS analysis of the *V. vinifera* samples, respectively. Each compound was identified through databases available to the laboratory and was associated with an MSI level of identification based on Sumner et al., 2007 [41]. GC-FID (for fatty acids) and UHPLC/UV-ESI-HRMS (other compounds) analyses were performed for all samples (SHE, SEA, SET, MWA, and MAE). Only the SHE and SEA samples contained compounds (fatty acids) detected by GC-FID analysis. For the SET, MWA, and MAE samples, UHPLC/UV-ESI-HRMS analysis showed the presence of (poly)phenolic compounds, which were not found in SHE and SEA.

In Table 4, Table 5, Table 6, Table 7 and Table 8, it is possible to observe the differences between the samples, both in terms of the bioactive composition and relative abundance of the various components. The fatty acids investigated were found only in the extracts where organic solvents with lower polarity had been used (hexane and ethyl acetate), while there was no trace of them in the other extracts. As reported in Table 4, in the SEA sample, the fatty acid component was mainly due to the presence of linoleic acid (68.48%), cis-9-oleic acid (16.38%), palmitic acid (8.28%), and stearic acid (4.30%); in the SHE sample, the fatty acid component was mainly due to the presence of the same components in different percentage abundances, i.e., linoleic acid (56.77%), cis-9-oleic acid (18.19%), palmitic acid (9.06%), and stearic acid (3.97%), and to the exclusive presence of myristoleic acid (0.20%) other than to the presence of an unknown compound with a percentage abundance of 9.30% that will be investigated in future work.

**Figure A1:**
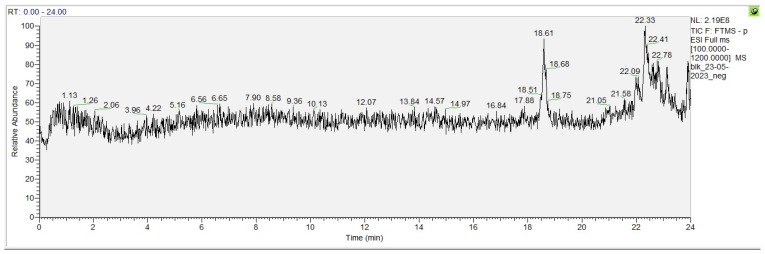
Chromatogram of blank by UHPLC/UV-ESI-HRMS analysis: negative polarity.

**Figure A2:**
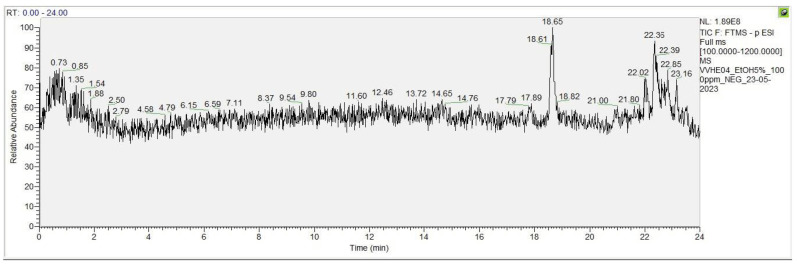
Chromatogram of SHE sample by UHPLC/UV-ESI-HRMS analysis: negative polarity.

**Figure A3:**
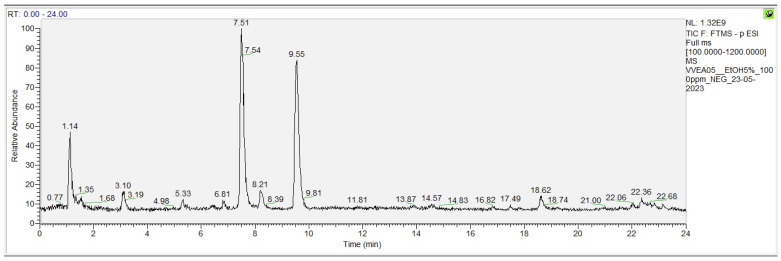
Chromatogram of SEA sample by UHPLC/UV-ESI-HRMS analysis: negative polarity.

**Figure A4:**
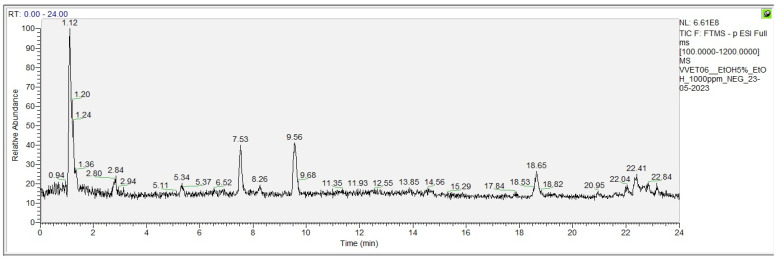
Chromatogram of SET sample by UHPLC/UV-ESI-HRMS analysis: negative polarity.

**Figure A5:**
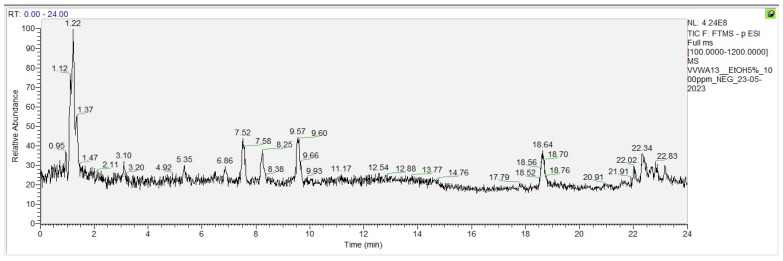
Chromatogram of MWA sample by UHPLC/UV-ESI-HRMS analysis: negative polarity.

**Figure A6:**
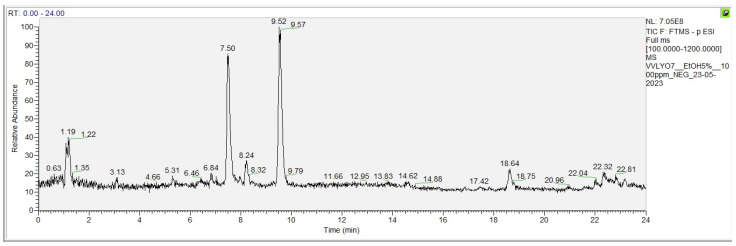
Chromatogram of MAE sample by UHPLC/UV-ESI-HRMS analysis: negative polarity.

**Figure A7:**
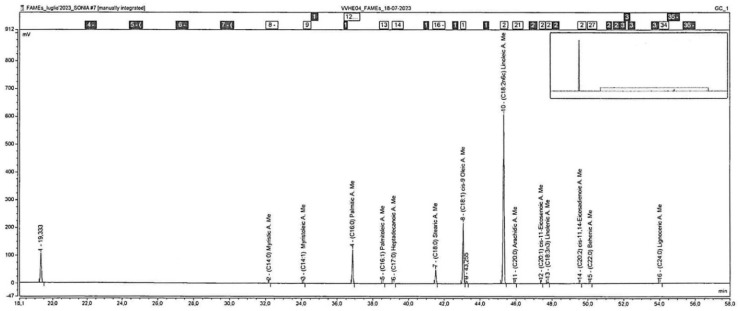
Chromatogram of SHE sample by GC-FID analysis.

**Figure A8:**
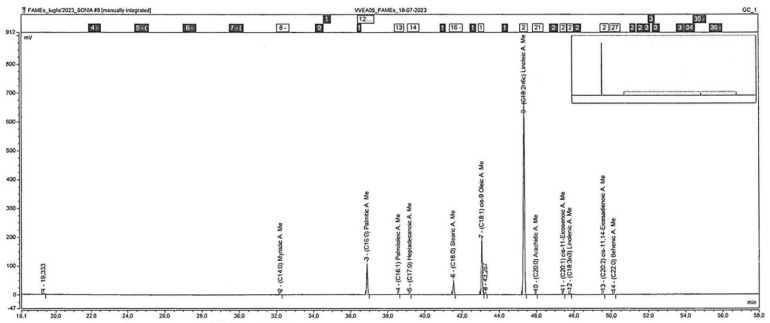
Chromatogram of SEA sample by GC-FID analysis.

Different bioactive compounds were found in all samples except for SHE. As reported in Table 5, in the SEA sample, the bioactive component was mainly due to the presence of (+) catechin (40.11%) and epicatechin (36.20%); in the SET sample (Table 6), the bioactive component was mainly due to the presence, along with the (+) catechin (13.66%) and epicatechin (19.26%) components, of glucose, citric acid, an unidentified dihexoside, and tartaric acid, with the total percentage of these components being 53.74%. In the MWA sample (Table 7), the bioactive component was mainly due to the presence of several components such as unidentified dihexoside and tartaric acid (34.53% tot.), malic acid (12.41%), (+) catechin (15.11%), and epicatechin (19.06%), as well as procyanidin dimer (9.94%). Finally, in the MAE sample (Table 8), the bioactive component was mainly due to the presence of (+) catechin (32.24%) and epicatechin (43.69%). More detailed data are reported in Appendix A (Figure A1, Figure A2, Figure A3, Figure A4, Figure A5, Figure A6, Figure A7 and Figure A8).

### 3.2. V. vinifera L. Effect on Cell Viability of HepG2 Cells

**Figure 1:**
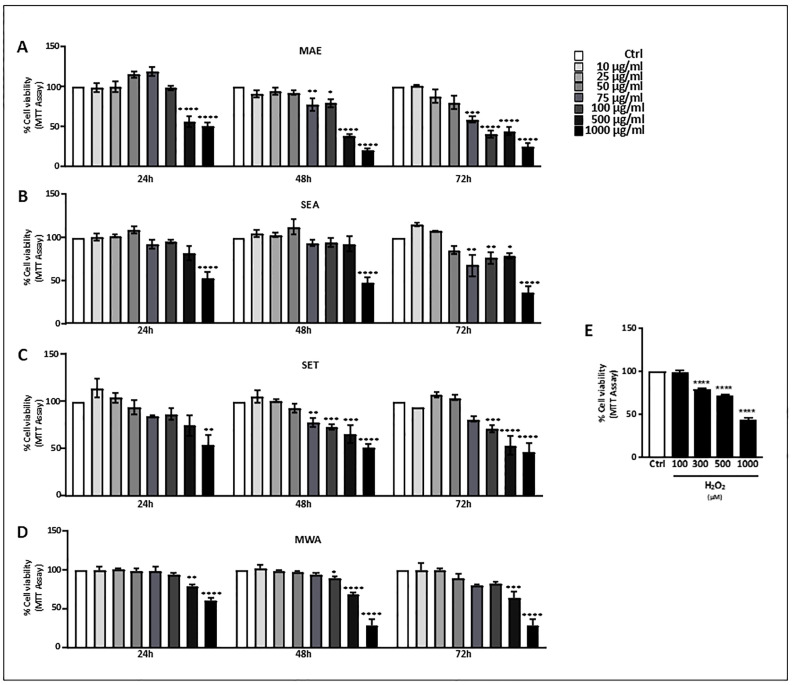
Cell viability of HepG2 cells exposed to increasing concentrations of *V. vinifera* L. seed ((**A**) MAE, (**B**) SEA, (**C**) SET, and (**D**) MWA) for 24, 48, and 72 h and determined by MTT assay. Results represent mean values of 6 independent experiments ± SE. * *p* < 0.05, ** *p* < 0.01, *** *p* < 0.001, and **** *p* < 0.0001 compared with control (cells incubated with vehicle only). (**E**) Evaluation of HepG2 cell viability after exposure to various concentrations of H_2_O_2_ (100, 300, 500, and 1000 μM) for 3 h. Values are the mean ± SEM of at least four independent experiments. * *p* < 0.05, ** *p* < 0.01, *** *p* < 0.001, and **** *p* < 0.0001.

To determine the optimal concentrations of *V. vinifera* L. seed extracts that can be used without causing cytotoxicity, the viability of HepG2 cells was tested by MTT assay. Each extract was used at different concentrations (10–1000 μg·mL^−1^) for 24, 48, and 72 h. As shown by the concentration–response curve, HepG2 cells exposed to seed extracts at concentrations greater than 100 μg·mL^−1^ had significantly impaired cell viability from 24 h, except for SEA, which showed cytotoxic effects at higher concentrations (1000 μg·mL^−1^) after 24 and even at 48 h (Figure 1B). With increasing exposure time, some of the extracts caused a decrease in cell viability already at a concentration of 100 μg·mL^−1^, such as MAE, SET, and MWA (Figure 1A,C,D), and at the lower concentration of 75 μg·mL^−1^ of SET and MAE after 48 h (Figure 1A,C). A longer exposure time further exacerbated the effects of each extract on cell viability in the concentration range from 75 μg·mL^−1^ to 1000 μg·mL^−1^. Since no effects on cell viability were observed after exposure to concentrations of 10 and 25 μg·mL^−1^ at all time points examined (Figure 1A,D), the concentration of 25 μg·mL^−1^ of *V. vinifera* L. seed extract was chosen for further analyses. These results suggested that the decreased levels of oxidative stress in subsequent experiments were not due to a decreased number of cells. The SHE extract was not considered for further analysis due to its high cytotoxicity.

### 3.3. V. vinifera L. Extracts Reduce H_2_O_2_-Induced Cell Death of HepG2 Cells

**Figure 2:**
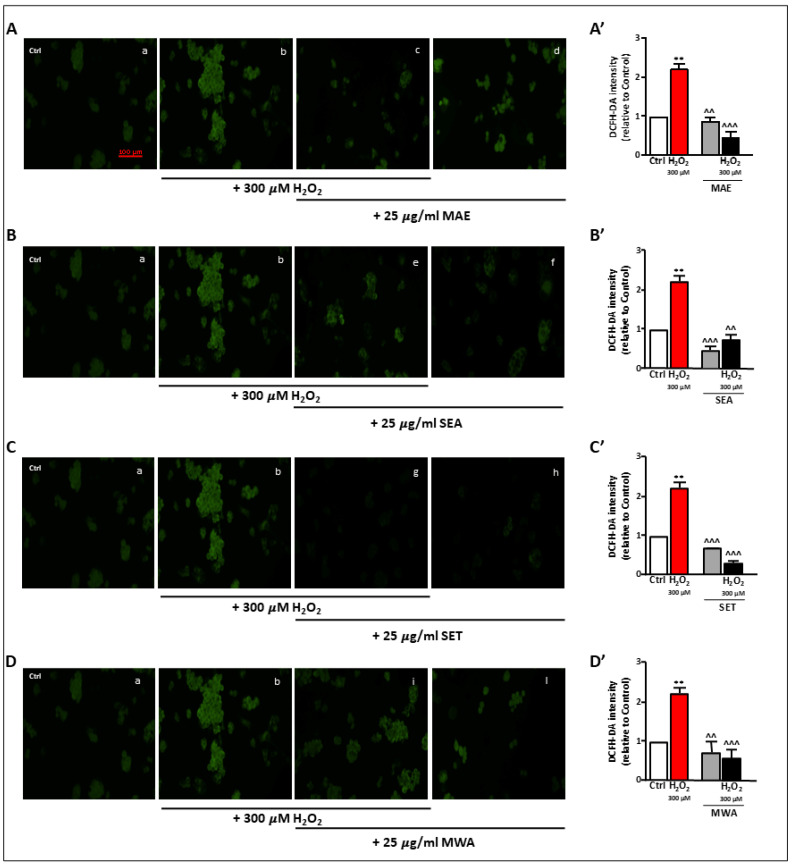
Effects of grape seed polyphenol extracts on intracellular ROS levels in HepG2 cells exposed to H_2_O_2_. (**A**–**D**) Representative images of HepG2 cells loaded with 2′-7′ dichlorodihydrofluorescein diacetate probe (DCFH-DA) to measure total intracellular ROS levels in the control and treated cells. (**A’**–**D’**) Bar graphs depicting quantification of fluorescence intensity expressed as fold increase of the control cells. ** *p* < 0.01 vs. Ctrl; ^^ *p* < 0.01, and ^^^ *p* < 0.001 vs. H_2_O_2_ statistical significance. Scale bar (**A**–**D**): 100 µm. Data expressed as mean ± SEM of three independent experimental sessions.

To analyze the effects of *V. vinifera* L. seed extracts on oxidative-stress injury, HepG2 cells were treated with increasing concentrations of H_2_O_2_ (100, 300, 500, and 1000 μM) for 3 h. MTT assay was performed to analyze the viability of the cells under H_2_O_2_ stimulation. As shown in Figure 1E, cell viability was affected by H_2_O_2_ treatments in a concentration-dependent manner, with 300 μM H_2_O_2_ being effective in decreasing cell viability by approximately 20%. Therefore, exposure to 300 μM H_2_O_2_ for 3 h was used to establish an oxidative stress injury model. We next evaluated the effect of *V. vinifera* L. seed extracts against H_2_O_2_-induced cell death. HepG2 cells treated with 300 μM H_2_O_2_ for 3 h were followed by 3 h exposure to 25 μg·mL^−1^ of each extract. As reported in Figure 2A–D, the SEA, SET, MAE, and MWA extracts were found to reduce the oxidative stress caused by H_2_O_2_. In contrast, when extracts were applied concomitantly to H_2_O_2,_ they did not elicit any cytoprotective effect.

### 3.4. V. vinifera L. Extracts Reduce H_2_O_2_-Induced Intracellular ROS Production in HepG2 Cells

**Figure 3:**
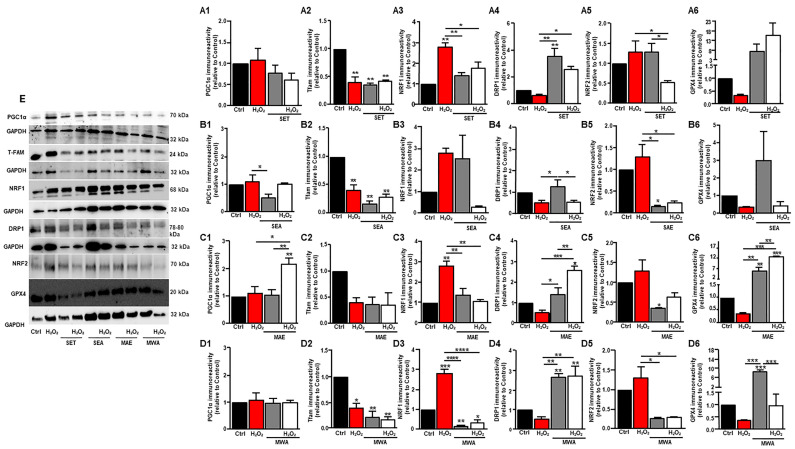
Effects of grape seed polyphenol extracts on protein expression of antioxidant defenses, mitochondrial biogenesis, and dynamics. Representative Western Blot (**E**) and relative bar graphs of (**A1**–**D1**) PGC1*α*, (**A2**–**D2**) Tfam, (**A3**–**D3**) NRF1, (**A4**–**D4**) Drp1, (**A5**–**D5**) NRF2, and (**A6**–**D6**) GPX4 in HepG2 cells exposed to H_2_O_2_ and then to extracts (SET, SEA, MAE, and MWA). * *p* < 0.05, ** *p* < 0.01, *** *p* < 0.001, and **** *p* < 0.0001.

One of the main factors involved in cell oxidative stress induced by H_2_O_2_ is an excessive ROS accumulation. Intracellular ROS was quantified by DCFH assay. As shown in Figure 2, compared with the control group, 300 μM H_2_O_2_ caused a significant increase in the rate of formation of ROS in HepG2 cells (more than 2-fold) (2.23 ± 0.15, *p* < 0.01). Next, 25 μg·mL^−1^ of each extract was used to assess their putative antioxidant effects. Interestingly, all extracts showed the capacity to reduce the H_2_O_2_-induced formation of intracellular ROS, with SET being the most effective (0.301 ± 0.04, *p* < 0.0002), as illustrated in Figure 2C. Meanwhile, to determine the role of the antioxidant enzymes for the effects by each extract against oxidative damage, the expressions of GPx4 were measured by Western Blot. The treatment with MAE, SET, and MWA markedly enhanced the GPx4 level, which was almost abrogated by treatment with H_2_O_2_ (Figure 3(A6,C6,D6,E)). Interestingly, MAE, SET, and MWA were all able to increase GPx4 levels, even in the absence of H_2_O_2_-induced oxidative stress (Figure 3(A6,C6,D6,E)). Accordingly, with its lower antioxidant capacity, SEA was not able to increase GPx4 levels (Figure 3(B6)). Endogenous ROS enhanced the expression of nuclear mitochondrial biogenesis markers NRF1 and NRF2, while the treatment with MAE, SET, and MWA in the presence of H_2_O_2_ markedly reduced NRF1 and NRF2 levels, reaching values not significantly different from the control ones, further supporting their capacity to act as antioxidants (Figure 3(A3,C3,D3,A5,C5,D5,E)).

### 3.5. Effect of V. vinifera L. Seed Extracts on Mitochondrial Activity

As mitochondrial morphology is critical for mitochondrial function, we investigated the quality of the extracts on DRP1⁄MFN2 as markers of fission and fusion balance. Notably, the levels of either DRP1 or MFN2 did not change in H_2_O_2_–HepG2-treated cells, while MAE, SET, and MWA extracts increased DRP1 (Figure 3(A4,C4,D4,E)) levels but were not effective on MFN2 ones, hence favoring fission as likely leading to mitophagy of damaged mitochondria. Mitochondrial fission is a highly regulated process by which mitochondria tubules divide into smaller fragments. This process is essential for mitochondrial quality control, as it allows for the removal of damaged or dysfunctional mitochondria via mitophagy. These results are indicative of a re-establishment of mitochondrial quality control by all the above extracts. By detecting PGC-1α levels, the master regulator of mitochondrial biogenesis, it was increased in H_2_O_2_–HepG2 cells following MAE exposure with unchanged Tfam levels (see Figure 2C). SET did not change PGC-1α and Tfam levels from those reached following H_2_O_2_ treatment (Figure 3(A1,A2,E)), while MWA did not change PGC-1α levels (Figure 3(D1,E)), although decreasing Tfam levels compared with those reached following H_2_O_2_ (Figure 3(D2,E)). SEA, which showed a lower capacity to diminish H_2_O_2_-induced ROS production, did not change any of the above-described markers (Figure 3(B1,B2,E)). Mitochondrial quality control resulted in the involvement of the antioxidant effects elicited by MAE, SET, and MWA, hence suggesting that the role of their polyphenols is at least partially due to their capacity to maintain mitochondrial biogenesis and mitophagy, which elevates mitochondrial efficiency, resulting in diminished ROS production.

## 4. Discussion

The seeds of *V. vinifera* are an important source of bioactive compounds that, when peculiarly extracted and functionalized according to their biochemical characteristics, can be used as nutraceutical and pharmacological products. Indeed, it is important to evaluate which extraction solvent is most effective on such a matrix. The °Bx parameter (Table 2) had a very low value for all samples: the refractometer analyzed the dissolved sugar content (dissolved solids content) as a percentage and returned very low percentage values. It is possible to assume that, given this value, the total contribution by weight is, as a percentage, attributed to the presence of (poly)phenolic or similar compounds and not to other compounds such as carbohydrates, lipids, or small peptides. These results are of great interest according to the literature [35,42,43,44] and previous works of our laboratory [35,45,46].

Based on what was obtained, we identified EA and ET as the best extractive solvents towards the (poly)phenolic compounds of the *V. vinifera* seed samples compared to WA and, even more so, HE. The MAE sample is comparable in terms of the TPC to the sum of the individual extractions shown in Table 3, with the same amount of extracted matrix and solvent used. This is because the solvent used manages to maximize the extraction of the (poly)phenolic compounds by achieving a total extraction comparable to the sum of the individual extractions, HE, EA, ET, and WA. This is an interesting result as, compared to studies by Di Meo et al., 2019 [32], the extracts produced can be understood as the purification of a total extract, which, thanks to the use of individual solvents, allows specific classes of bioactive compounds to be obtained, the effects of which can be specifically studied.

Furthermore, high antiradical activity (expressed as I%) was verified in the SET, MWA, and MAE extracts compared with the positive control (Trolox), with the percentages all being above 95%, while the activity was not as satisfactory for the SHE and non-SEA extracts. Compared with other studies in the literature, this activity appears to have been found for the first time for *V. vinifera* L. cv. Falanghina. Indeed, Yemis et al., 2007 [47], reported samples of extract from different Turkish white grape varieties, such as Muskule, Razaki, Emir, Hasandede, and Narince, and showed excellent antioxidant activity for the latter. Moreover, Keser et al., 2013 [48], tested the Turkish white grape variety Silfoni and showed lower ratios of the amount of extracted sample and extracting solvent.

### 4.1. Evaluation of LC-MS/MS and GC/FID Analysis

Comparing the available literature [26,28,49,50], the samples reported here show, depending on the extract considered, relative percentage amounts of fatty acids such as linoleic acid and cis-9 oleic acid. No work so far has focused specifically on obtaining fatty acids from white grapes seeds, counting even the Soxhlet technique itself and other techniques such as supercritical fluid extraction. To date, relatively few studies have been carried out specifically on Falanghina and more specifically on the bioactive compound content of its seeds. The fatty acids examined and linoleic acid and cis-9 oleic acid, as for Kapcsándi et al., 2021 [26], Guler et al., 2021 [28], and Yalcin et al., 2016 [49], always appear to be those with the highest relative percentages compared to the total percentage of fatty acids detected. Instead, Pérez-Navarro et al., 2019 [50], reported different relative percentages, being the relative percentages also important for palmitic acid and stearic acid. Such a difference may be due to various factors (not discussed in this work) that can be identified in the cultivar, in the type of substrate where the plants grow, the specific climate of the area, and other different factors that generate the metabolites produced by the plant within a great diversity (even if only the relative percentage of the components analyzed is considered). Also, in the case of bioactive compounds, especially in the case of catechins, the extracts are well represented. The presence of these flavanolic components [27,51,52,53,54] has been reported within the seeds and also in other parts of the plant. The same consideration can be made for the remaining compounds found, such as citric acid, tartaric acid, gallic acid, and malic acid.

### 4.2. Cell Assay

This study reported for the first time the characterization of the antioxidant capacity of the seed extracts from white grapes, together with the assessment of their functional properties on mitochondrial quality control in HepG2 hepatocytes following exposure to H_2_O_2_. Our findings further confirmed that the polyphenols content is capable of imparting beneficial effects by reducing ROS intracellular levels. The mitochondrial quality control process accounts for regulation of mitochondrial quantity and quality, hence maintaining mitochondrial homeostasis in cells [55]. In such a process, a key mechanism in the dynamic control and repair of mitochondrial quality is the mitochondrion fission and fusion [56] which, together with mitophagy and mitochondrial biosynthesis, promotes the degradation and renewal of mitochondria, respectively. A functional bioactive extract may lead to safeguard mitochondrial defense, repair, and removal and, thus, may activate mechanisms which play indispensable roles in determining cell fate by maintaining the functional homeostasis of mitochondria. Indeed, an imbalance in mitochondrial dynamics results in an alteration of the mitochondrial number, morphology, and functioning, leading to the development of various diseases, including cancer. Fission is essential for maintaining the mitochondrial number and proper distribution in the daughter cells, while fusion ensures optimal mitochondrial activity by allowing the exchange of contents between fusing mitochondria to meet different physiological needs [56]. Data reported here suggest that MAE, SET, and MWA increase mitophagy, which is part of the essential quality control mechanism. Indeed, the involvement of mitochondrial quality control into the antioxidant effects elicited by MAE, SET, and MWA is in favor of the role played by their polyphenols, at least partially due to their capacity to maintain mitochondrial efficiency, resulting in diminished ROS production and, hence, further supporting their capacity to act as antioxidants. Notably, the lower antioxidant capacity of SEA was associated with a lack of effects on the mitochondrial quality control process. Taken together, these findings introduce MAE, SET, and MWA as compelling candidates capable of imparting beneficial antioxidant effects while maintaining mitochondria quality control and opening a new way of determining the quality of plant-derived products that is more closely linked to the biological activity of a product and its potential effectiveness. Future work should focus on generating data to further validate this testing methodology.

## 5. Conclusions

Quantification of the polyphenolic compounds in *V. vinifera* seed extracts is essential to elucidate the effects of the bioactive compounds as a mix and to investigate the mechanistic basis of the observed effects. Mitochondrial analysis and associated chemical analysis methods propose an innovative way of determining the quality of plant by-product extracts that is more closely linked to their biological activity and potential effectiveness. Taken together, the observed antioxidant and mitochondria-affecting properties of the winery by-products suggest that they may be interesting sources of bioactive compounds acting as effective antioxidant agents and may indicate a promising role for the protection and regulation of metabolic pathways under oxidative stress conditions. The results reported here support *V. vinifera* bioprospection based on the circular economy concept toward the valorization of wine-plant by-products providing opportunities to develop value-added products. In addition, a reuse or alternative application of by-products in alternative alimentary or supplement products is a great way to validate the Sustainable Development Goals of 2030 Agenda.

## Figures and Tables

**Table 1:** Codification of extract samples subjected to analysis.

Sample ID	Extraction Solvent	Ratio (%)
SHE	Hexane	100
SEA	Ethyl acetate	100
SET	Ethanol	100
MWA	Water–Acetone	60:40
MAE	Ethanol–Ethyl acetate	80:20

S = Soxhlet extraction technique; M = maceration extraction technique; HE = extract in hexane solvent; EA = extraction in ethyl acetate solvent; ET = extract in absolute ethanol solvent; WA = extract in solution mixture of water (bidistilled and deionized) and acetone (6:4 ratio, *v/v*); AE = extract in solution mixture of ethanol and ethyl acetate (8:2 ratio, *v/v*).

**Table 2:** Specific gravity, dry residue, and extract value of the different *V. vinifera* extracts. Values are presented as mean (triplicate) ± SD.

ID	Brix (°Bx)	Residue (g∙L^−1^)	Extract $(mg\cdot g_{seed}^{-1}$ D.W.)
SHE	0.2 ± 0.1	0.6103 ± 0.0024	17.44 ± 3.90
SEA	0.2 ± 0.1	1.402 ± 0.0052 *	40.04 ± 2.70 *
SET	1.2 ± 0.1 *	1.403 ± 0.0019 *	40.08 ± 3.30 *
MWA	1.1 ± 0.1 *	1.621 ± 0.0021 **	46.32 ± 1.30 ***
MAE	1.2 ± 0.1 *	1.595 ± 0.0035 **	42.76 ± 4.20 **

D.W. = dried weight; *, **, *** = Asterisks indicate significant differences for parameters between extraction methods at *p* < 0.05, *p* < 0.01, *p* < 0.001 respectively.

**Table 3:** Total phenolic compound content (TPC) and DPPH radical scavenging activity (I%) of the different *V. vinifera* extracts. Values are presented as mean (triplicate) ± SD.

ID	TPC (mg GAE·g^−1^)	% Free Radical Inhibition (I%)
SHE	0.40 ± 0.02	65.79 ± 0.94 *
SEA	18.12 ± 0.41 **	51.66 ± 0.47
SET	18.07 ± 0.88 **	98.32 ± 0.12 **
MWA	14.17 ± 0.51 *	96.31 ± 0.02 **
MAE	34.02 ± 2.35 ***	96.56 ± 0.37 **

GAE expresses the equivalents of gallic acid. Trolox standard solution 50 µL (100 µg·mL^−1^) was used as the positive control with I% = 95.03 ± 0.22%. *, **, *** = Asterisks indicate significant differences for parameters between extraction methods at *p* < 0.05, *p* < 0.01, *p* < 0.001 respectively.

**Table 4:** Composition of fatty acids (FAMEs) * reported as relative percentages of peaks area (%) identified by GC-FID.

	SHE		SEA	
Assigned Identity	Rt (min)	Area (%)	Rt (min)	Area (%)
Unknown	19.333	9.30 ± 0.24	19.333	0.49 ± 0.08
Myristic acid	32.210	0.13 ± 0.03	32.215	0.09 ± 0.02
Myristoleic acid	34.133	0.20 ± 0.06	-	-
Palmitic acid	36.882	9.06 ± 0.19	36.883	8.28 ± 0.11
Palmitoleic acid	38.573	0.28 ± 0.5	38.578	0.15 ± 0.04
Heptadecanoic acid	39.195	0.08 ± 0.1	39.198	0.06 ± 0.01
Stearic acid	41.530	3.97 ± 0.29	41.543	4.30 ± 0.15
cis-9 Oleic acid	43.070	18.19 ± 0.20	43.080	16.38 ± 0.18
Unknown	43.255	0.83 ± 0.11	43.267	0.78 ± 0.13
Linoleic acid	45.340	56.77 ± 0.31	45.358	68.48 ± 0.21
Arachidic acid	45.973	0.17 ± 0.02	45.982	0.17 ± 0.03
cis-11-Eicosenoic acid	47.427	0.20 ± 0.08	47.437	0.18 ± 0.02
Linolenic acid	47.792	0.59 ± 0.13	47.797	0.55 ± 0.10
cis-11,14-Eicosadienoic acid	49.567	0.05 ± 0.01	49.582	0.05 ± 0.01
Behenic acid	50.148	0.08 ± 0.02	50.142	0.04 ± 0.01
Lignoceric acid	54.035	0.09 ± 0.01	54.035	0.09 ± 0.01

* Data expressed as means and standards deviations (±SD) of three observations.

**Table 5:** Metabolites identified in SEA sample by UHPLC/UV-ESI-HRMS analysis reported as relative percentages of peaks area (%) *.

Rt (min)	[M − H]^−^	[M + H]^+^	Main MS/MS Fragments (*m*/*z*)	Assigned Identity	Area (%)	MSI Status ^a^
1.14	179.0552	-	89, 71, 59	glucose	12.53 ± 0.28	2
1.14	215.0318	-	89, 71, 59	citric acid		2
3.10	169.01134	-	125	gallic acid	4.33 ± 0.41	2
5.31	331.0670	-	169, 125	monogalloyl-glucose	0.21 ± 0.03	2
6.81	577.1351	-	407, 289, 245, 125	procyanidindimer	1.65 ± 0.18	2
7.49	289.0716	291.0855	245, 203, 137, 125, 109 (pos. 139, 123, 147, 165)	(+) catechin	40.11 ± 0.36	2
8.21	577.1351	-	407, 289, 245, 125	procyanidindimer	4.97 ± 0.23	2
9.55	289.0716	291.0855	245, 203, 137, 125, 109 (pos. 139, 123, 147, 165)	epicatechin	36.20 ± 0.25	2

^a^ MSI level of identification based on Sumner et al., 2007 [41]. * Data expressed as means and standards deviations (±SD) of three observations.

**Table 6:** Metabolites identified in SET sample by UHPLC/UV-ESI-HRMS analysis reported as relative percentages of peaks area (%) *.

Rt (min)	[M − H]^−^	[M + H]^+^	Main MS/MS Fragments (*m*/*z*)	Assigned Identity	Area (%)	MSI Status ^a^
1.12	179.0552	-	89, 71, 59	glucose	53.74 ± 0.31	2
1.12	215.0318	-	89, 71, 59	citric acid		2
1.12	387.1142	-	341, 179, 119, 113, 89	dihexoside		2
1.12	149.0080	-	103, 87, 72, 59	tartaric acid		2
2.84	169.01134	-	125	gallic acid	6.52 ± 0.22	2
5.31	331.0670	-	169, 125	monogalloyl-glucose	3.68 ± 0.15	2
7.53	289.0716	-	245, 203, 137, 125, 109	(+) catechin	3.14 ± 0.09	2
8.26	577.1351	-	407, 289, 245, 125	procyanidindimer	13.66 ± 0.37	2
9.56	289.0716	291.0855	245, 203, 137, 125, 109 (pos. 139, 123, 147, 165)	epicatechin	19.26 ± 0.20	2

^a^ MSI level of identification based on Sumner et al., 2007 [41]. * Data expressed as means and standards deviations (±SD) of three observations.

**Table 7:** Metabolites identified in MWA sample by UHPLC/UV-ESI-HRMS analysis reported as relative percentages of peaks area (%) *.

Rt (min)	[M − H]^−^	[M + H]^+^	Main MS/MS Fragments (*m*/*z*)	Assigned Identity	Area (%)	MSI Status ^a^
1.12	179.0552	-	89, 71, 59	glucose	1.66 ± 0.14	2
1.12	195.0502	-	159, 129, 99, 75	gluconic acid		2
1.12	215.0318	-	89, 71, 59	citric acid		2
1.22	387.1142	-	341, 179, 119, 113, 89	dihexoside	34.53 ± 0.29	2
1.22	149.0080	-	103, 87, 72, 59	tartaric acid		2
1.36	133.0130	-	115, 71	malic acid	12.41 ± 0.33	2
3.10	169.01134	-	125	gallic acid	3.88 ± 0.11	2
5.34	331.0670	-	169, 125	monogalloyl-glucose	3.40 ± 0.16	2
7.50	289.0716	-	245, 203, 137, 125, 109	(+) catechin	9.94 ± 0.26	2
8.25	577.1351	-	407, 289, 245, 125	procyanidindimer	15.11 ± 0.07	2
9.57	289.0716	291.0855	245, 203, 137, 125, 109 (pos. 139, 123, 147, 165)	epicatechin	19.06 ± 0.12	2

^a^ MSI level of identification based on Sumner et al., 2007 [41]. * Data expressed as means and standards deviations (±SD) of three observations.

**Table 8:** Metabolites identified in MAE sample by UHPLC/UV-ESI-HRMS analysis reported as relative percentages of peaks area (%) *.

Rt (min)	[M − H]^−^	[M + H]^+^	Main MS/MS Fragments (*m*/*z*)	Assigned Identity	Area (%)	MSI Status ^a^
1.12	179.0552	-	89, 71, 59	glucose	5.43 ± 0.36	2
1.12	215.0318	-	89, 71, 59	citric acid		2
1.22	387.1142	-	341, 179, 119, 113, 89	dihexoside	8.10 ± 0.21	2
3.13	169.01134	-	125	gallic acid	1.57 ± 0.10	2
5.31	331.0670	-	169, 125	monogalloyl-glucose	1.78 ± 0.16	2
6.81	577.1351	-	407, 289, 245, 125	procyanidindimer	1.74 ± 0.22	2
7.50	289.0716	-	245, 203, 137, 125, 109	(+) catechin	32.24 ± 0.24	2
8.23	577.1351	-	407, 289, 245, 125	procyanidindimer	5.45 ± 0.12	2
9.55	289.0716	291.0855	245, 203, 137, 125, 109 (pos. 139, 123, 147, 165)	epicatechin	43.69 ± 0.30	2

^a^ MSI level of identification based on Sumner et al., 2007 [41]. * Data expressed as means and standards deviations (±SD) of three observations.

## Data Availability

Data is contained within the article.
